# A Prospective Interventional Study Comparing a Multimodal Intervention Incorporating Behavior, Exercise, Nutrition, Medication, and Posture With Exercise and Medication in Patients With Chronic Nonspecific Low Back Pain

**DOI:** 10.7759/cureus.117483

**Published:** 2026-10-05

**Authors:** Sameep H Koshti, Amit Soni, Himanshu Champaneri, Deepak K Bhangale, Amit Agrawal, Sudhir Dubey

**Affiliations:** 1 Neurosurgery, Park Hospital, Panipat, IND; 2 Neurorehabilitation, Medanta - The Medicity, Gurgaon, IND; 3 Neurosurgery, Max Hospital, Gurgaon, IND; 4 Neurosurgery, Medanta - The Medicity, Gurgaon, IND; 5 Neurosurgery, All India Institute of Medical Sciences, Bhopal, IND

**Keywords:** behavioral therapy, chronic nonspecific low back pain, exercise therapy, holistic approach, nutrition optimization

## Abstract

Background and objectives

Chronic nonspecific low back pain (CNLBP) is a leading cause of disability, and its management remains a significant challenge. While non-pharmacological interventions like exercise are recommended, the optimal treatment strategy is unclear. This study compared the effectiveness of a comprehensive, five-modality holistic program (behavior therapy, exercise, nutrition optimization, drugs, and posture training) with a standard two-modality intervention (exercise and drugs) for patients with CNLBP.

Methods

This prospective interventional study included 123 patients with CNLBP. Participants were randomly allocated to either a two-modality intervention (Group 1; n = 60) or a five-modality holistic program (Group 2; n = 63). The primary outcome was the between-group difference in pain (Visual Analogue Scale, VAS) at the six-month follow-up. Secondary endpoints, including changes in anxiety scores and analysis by pain location, were treated as exploratory.

Results

After six months, both groups showed clinical improvement. However, the five-modality group (Group 2) demonstrated statistically significant and superior improvements in pain compared to the two-modality group (Group 1). The between-group difference in mean improvement for the VAS score (3.65 vs. 1.55) was statistically significant (p < 0.001). Subgroup analysis revealed that patients with higher baseline anxiety showed significantly greater improvement with the holistic approach.

Conclusions

Findings from the current study support the adoption of a holistic, multidisciplinary strategy for the management of CNLBP. The integration of behavior therapy, nutrition optimization, and posture training alongside conventional exercise and medication provides a superior, long-term therapeutic approach. A comprehensive, five-modality treatment program is significantly more effective than a standard two-modality intervention for improving pain in patients with chronic nonspecific spinal pain.

## Introduction

Chronic nonspecific low back pain (CNLBP) is a complex, multifactorial condition of global public health concern [1]. CNLBP is characterized by persistent discomfort in the lower back, lumbosacral, and sacroiliac regions lasting for more than three months [2,3]. It is one of the leading causes of disability, with its economic cost accounting for more than $100 billion annually in the USA alone [4-6]. In addition to financial losses, CNLBP can lead to physical and mental disability, including depression, anxiety, psychosis, and sleep deprivation [7-9]. Compounding this issue, in the majority of cases, the underlying cause is not known [10,11]. Current clinical guidelines recommend combining pharmacological and non-pharmacological interventions [12,13]. Commonly used medications include nonsteroidal anti-inflammatory drugs (NSAIDs) and neuropathic agents (e.g., Gabapentin or tricyclic antidepressants), though these are often insufficient as monotherapy [7,14]. Physical interventions, particularly exercise therapy, are a cornerstone of treatment [15,16]; specifically, core stabilization (focusing on the deep multifidus and transversus abdominis) and mind-body interventions (e.g., Pilates, Yoga, Tai Chi) have demonstrated benefits in improving functional capacity and modulating pain [11,14]. However, despite these options, outcomes for CNLBP remain uncertain [5,16], suggesting a critical gap in management. It is important to differentiate CNLBP from specific spinal pathologies (e.g., stenosis or disc herniation with radiculopathy), which were excluded in this study through clinical and radiological assessment [10,17]. While the term CNLBP is used, our cohort includes patients with combined cervical and lumbar discomfort, representing a broader presentation of chronic nonspecific spinal pain. It is important to differentiate CNLBP from specific spinal pathologies (e.g., stenosis or disc herniation with radiculopathy), which were excluded in this study through clinical and radiological assessment. While the term CNLBP is used, our cohort includes patients with combined cervical and lumbar discomfort, representing a broader clinical presentation of chronic nonspecific spinal pain. The primary hypothesis of this study is that a comprehensive, five-modality intervention will yield statistically superior pain relief compared to a standard two-modality intervention, with pain intensity at the six-month follow-up defined as the primary endpoint. Therefore, this prospective interventional study investigates whether a holistic, five-modality program (behavior therapy, exercise, nutrition, drugs, and posture) can produce superior outcomes compared to a standard two-modality intervention (exercise and drugs) for patients with chronic nonspecific spinal pain.

## Materials and methods

Study design and participants

This study utilized a prospective cohort design with follow-up data collection conducted at the six-month mark. The study was conducted at the Neurosurgery Outpatient Department of Medanta Hospital, Gurgaon, between June 2017 and May 2019. For the purpose of this analysis, baseline data were collected prospectively, while follow-up information was completed via outpatient visits and retrospective review of electronic hospital records. The study enrolled patients diagnosed with nonspecific chronic back pain who were candidates for nonoperative management. The exclusion criteria included the presence of "red flags" suggesting serious conditions (e.g., age <20, neurological deficits, history of cancer, significant trauma) or any specific cause of back pain identified through detailed history, clinical examination, and radiological investigations (X-ray/MRI). All patients were comprehensively screened to exclude individuals with specific pathologies. After exclusions, a final sample of 123 patients was included in the analysis.

Interventions and group allocation

Following diagnosis and enrollment, participants were sequentially allocated to one of two treatment groups using a simple randomization process. Group 2 (n = 63) received a five-modality intervention, which included behavior therapy, exercise, nutrition optimization, drugs, and posture training. The interventions were delivered by a senior neurosurgeon and skilled physiotherapists. Behavioral therapy was utilized to address common psychosocial barriers to recovery, such as fear-avoidance beliefs and maladaptive pain-coping strategies, while psychological counseling was provided only for participants identified with significant depressive symptoms. The exercise intervention was standardized for all participants, with progression adapted to individual physical tolerance. The rationale for this selection was to address the core muscle atrophy and postural mechanical strain frequently associated with chronic nonspecific spinal pain [18,19]. The exercise protocol consisted of three primary components: (1) core stabilization, targeting the transversus abdominis and deep multifidus muscles to improve spinal segmental stability; (2) lumbar extensor strengthening, aimed at improving the strength and endurance of the posterior chain to reduce spinal load; and (3) postural correction, aimed at optimizing alignment to prevent re-injury and decrease ongoing mechanical strain. Posture training and exercises were taught and reinforced with pictorial leaflets for a home program. Nutrition optimization involved a dietary assessment to calculate daily protein, carbohydrate, and fat intake, followed by a personalized diet plan that included a high-protein diet, intended to support muscle mass maintenance and recovery, specifically addressing the risk of sarcopenia, a known limiting factor in rehabilitation for chronic pain patients [20,21]. Pharmacological treatment included analgesics, Gabapentin, and/or Nortriptyline as needed for pain relief in both groups. The summary of the intervention protocol is provided in Table 1.

**Table 1:** Summary of multimodal intervention protocol

Modality	Frequency/duration	Content/focus
Exercise	3x/week, 45-min sessions	Core stabilization, lumbar extensor strengthening, postural alignment
Behavioral therapy	4 sessions (weeks 0, 4, 8, 12)	Fear-avoidance, maladaptive coping, pain-related distress
Nutrition	Baseline + monthly review	High-protein diet (target 1.2 g/kg body weight) to address sarcopenia
Posture	Daily home program	Neutral spine training, ergonomic education, home exercise leaflets
Medication	As needed	Analgesics, Gabapentin, or Nortriptyline (monitored via patient diary)

Data collection and outcome measures

Data were collected in a predesigned proforma at a baseline visit and at a six-month follow-up appointment. Follow-up data were collected at the outpatient department (OPD) or via telephone for patients who could not attend the OPD. The primary outcome measure was the change in pain, assessed using the Visual Analogue Scale (VAS). Additionally, a standardized psychometric assessment for anxiety was administered at both time points.

Statistical analysis

Statistical analysis was performed using IBM SPSS Statistics for Windows, Version 22.0 (Released 2013; IBM Corp., Armonk, NY, USA). Quantitative data were presented as means and standard deviations, while categorical data were expressed as absolute numbers and proportions. To account for the observed baseline imbalance in VAS scores between the treatment groups, an analysis of covariance (ANCOVA) was employed, with baseline VAS scores used as the covariate for the estimate of the treatment effect on pain reduction at the six-month follow-up. For all analyses, a p-value of <0.05 was considered statistically significant. For the interpretation of clinical outcomes, a change in VAS score of ≥1.5 to 2.0 points was considered the threshold for a Minimal Clinically Important Difference (MCID) in pain reduction.

## Results

A total of 130 patients with chronic nonspecific spinal pain were enrolled in the study. From this initial cohort, seven cases (5.4%) were excluded from the final analysis: four (3.1%) were lost to follow-up, while three (2.3%) required more invasive intervention (two surgical discectomies and one root block injection). This resulted in a per-protocol sample of 123 patients for data analysis. Participants were allocated into two intervention arms: Group 1 (two modalities, n = 60) and Group 2 (five modalities, n = 63). The baseline demographic and clinical characteristics of the study participants are detailed in Table 2.

**Table 2:** Baseline demographics and clinical characteristics of the study participants *Statistically significant baseline difference observed (p < 0.05).

Characteristic	Overall (N = 123)	Group 1 (n = 60)	Group 2 (n = 63)	p-value
Age (years), mean ± SD	41.45 ± 8.91	42.28 ± 8.40	40.66 ± 9.36	0.317
Sex, n (%)				
Male	83 (67.5)	41 (68.3)	42 (66.7)	0.854
Female	40 (32.5)	19 (31.7)	21 (33.3)	
Height (m), mean ± SD	1.64 ± 0.06	1.65 ± 0.06	1.63 ± 0.06	0.093
Weight (kg), mean ± SD	68.04 ± 9.62	69.06 ± 9.91	67.06 ± 9.31	0.25
BMI (kg/m ²), mean ± SD	25.14 ± 3.49	25.23 ± 3.81	25.05 ± 3.20	0.78
Pain duration (years), mean ± SD	2.71 ± 2.40	2.65 ± 2.30	2.77 ± 2.50	0.789
Pain location, n (%)				
Cervical	4 (3.3)	2 (3.3)	2 (3.2)	0.985
Lumbar	35 (28.5)	17 (28.3)	18 (28.6)	
Cervical + lumbar	84 (68.3)	41 (68.3)	43 (68.2)	
Baseline anxiety, mean ± SD	10.41 ± 8.16	9.71 ± 6.77	11.07 ± 9.35	0.359
Baseline VAS, mean ± SD	6.55 ± 1.10	5.92 ± 1.05	7.16 ± 1.15	<0.05*

The mean age of the cohort was 41.45 ± 8.91 years, with a majority of male participants (67.5%). The mean body mass index (BMI) was 25.14 ± 3.49 kg/m². The most prevalent pain location was a combination of cervical and lumbar pain (68.3%), and the average duration of symptoms was 2.71 ± 2.4 years. At baseline, the two intervention groups were comparable in terms of age, anthropometric measures, and anxiety scores, with no statistically significant differences observed. However, given a baseline imbalance in pain severity (mean VAS 5.92 vs. 7.16), an ANCOVA was employed for the primary comparison, using baseline VAS as a covariate. After the six-month intervention period, the cohort as a whole showed significant clinical improvement. The mean VAS score decreased from 6.55 to 3.42. A comparison between the intervention groups revealed that Group 2, which received the five-modality treatment, experienced significantly greater improvements in pain compared to Group 1. After adjustment for baseline scores, the difference in the mean improvement for the VAS score (3.65 vs. 1.55) remained statistically significant (p < 0.001), as shown in Table 2. Subgroup analyses provided additional insights. There were no significant differences in pain improvement between male and female participants. Analysis by pain location showed that improvements in VAS varied significantly depending on whether the pain was cervical, lumbar, or combined. Correlation analysis showed that higher baseline anxiety scores were significantly correlated with greater improvements in VAS (p = 0.005). Neither age, BMI, nor the duration of back pain showed a significant correlation with improvements in pain scores.

## Discussion

The primary finding of this prospective interventional study is that a five-modality holistic approach, comprising behavior therapy, exercise, nutrition optimization, drugs, and posture training, resulted in statistically significant and greater improvements in pain compared to a two-modality approach of exercise and drugs alone. This finding suggests that a multidisciplinary strategy provides superior outcomes for patients with CNLBP. This is in agreement with previous studies showing that multidisciplinary approaches, including physical therapy, provide long-term improvement in pain status in patients with CNLBP [22,23].

Our results are consistent with literature supporting exercise as a cornerstone of CNLBP management, primarily because exercise is a meaningful way to increase functional capacity [21]. Numerous studies have shown that exercise in its various forms, including Pilates, yoga, core stabilization, and Tai Chi, can significantly improve pain levels [14,24]. The exercise component of our intervention, which focused on core stabilization and postural exercises, builds upon findings that demonstrate a close correlation between CNLBP and core muscles, particularly the deep multifidus and transversus abdominis [18]. Furthermore, musculoskeletal strengthening of the lumbar extensors is a key component in reducing pain [19], and our results reinforce that improving core function is a key element in treating CNLBP [25]. The significant improvements observed in our five-modality group may be attributed to the combined effect of the added interventions, and we hypothesize that these components may act synergistically, suggesting that a multi-faceted approach should be adopted when managing this complex condition. Further research is required to isolate the independent contribution of each modality to the overall clinical outcome.

Specific posture training complemented the exercise component by aiming to reduce ongoing mechanical strain and prevent re-injury, a factor that likely contributed to the superior results seen in Group 2. This approach is supported by evidence that posture-focused exercise helps strengthen core muscles of the lumbar region and promotes a neutral spine [26]. This targeted strengthening is important as even after the spontaneous resolution of symptoms, a lack of exercise can lead to persistent lumbar muscle atrophy [19].

In our study, we included nutrition optimization to address the issue of sarcopenia, defined as a loss of muscle mass, which is a known limiting factor for physical activity [20]. Our study, with a mean age of 41, suggests this is also a consideration for middle-aged individuals. We hypothesize that patients benefited from the personalized diet plan and high-protein intake, which supported the physiological demands of the exercise protocol.

The co-prevalence of pain and mental distress is known to affect chronic musculoskeletal conditions and lead to poorer outcomes [27,28]. The inclusion of behavioral therapy in the current study helped to address the psychological aspects of chronic pain. Our finding that patients with higher baseline anxiety scores showed significantly greater improvements following the five-modality intervention suggests that addressing these psychosocial factors is an important part of a comprehensive management protocol. This idea is also supported by Vitoula et al. [29], who highlighted that cognitive behavioral therapy (CBT) was effective in patients with CLBP, especially in reducing pain perception and helping patients improve their outcomes.

Limitations

The present study has several limitations. Similar to many trials involving exercise interventions, blinding of both participants and clinicians was not possible, which may introduce a risk of performance bias [14]. While our study protocol was prospectively designed, follow-up data collection involved a combination of outpatient visits and retrospective review of hospital records, which may introduce potential information bias. Compliance with the prescribed home programs was assumed but not formally measured, and medication administration was managed based on clinical as-needed protocols rather than standardized dosing, which limits our ability to quantify specific drug exposure between the two groups. Additionally, while pain was significantly reduced, this study did not report on standardized functional disability indices, nor was it formally registered in a clinical trial registry. Finally, this was a single-center study, and therefore, the findings may not be fully generalizable to a broader population. Additionally, our findings are limited by a baseline imbalance in pain severity and the potential for attention-based non-specific effects, as the holistic group received more contact time. Given our six-month follow-up, our results represent mid-term therapeutic outcomes rather than long-term sustained benefits. To address these limitations, future multicenter studies incorporating standardized drug dosing, adherence monitoring, and functional scales are recommended [16,20].

## Conclusions

The findings from the current study suggest that a comprehensive five-modality treatment program is an effective multidisciplinary strategy for reducing pain in patients with chronic nonspecific spinal pain. The comprehensive plan integrated behavior therapy, nutrition optimization, and posture training along with conventional exercise and appropriate medications. While this multimodal package provides a useful approach for mid-term pain management, further research is required to understand the independent contribution of each modality to the overall clinical outcome. Based on our findings, a comprehensive, multidisciplinary management strategy should be considered as a possible therapeutic option for the management of chronic nonspecific spinal pain.

## References

[1] van Tulder M, Becker A, Bekkering T (2006). Chapter 3. European guidelines for the management of acute nonspecific low back pain in primary care. Eur Spine J.

[2] Zhao K, Zhang P, Li H, Li L (2025). Exercise prescription for improving chronic low back pain in adults: a network meta-analysis. Front Public Health.

[3] Maher C, Underwood M, Buchbinder R (2017). Non-specific low back pain. Lancet.

[4] Guo HR, Tanaka S, Halperin WE, Cameron LL (1999). Back pain prevalence in US industry and estimates of lost workdays. Am J Public Health.

[5] Katz JN (2006). Lumbar disc disorders and low-back pain: socioeconomic factors and consequences. J Bone Joint Surg Am.

[6] Rubin DI (2007). Epidemiology and risk factors for spine pain. Neurol Clin.

[7] Kalso E, Edwards JE, Moore AR, McQuay HJ (2004). Opioids in chronic non-cancer pain: systematic review of efficacy and safety. Pain.

[8] Melloh M, Röder C, Elfering A (2008). Differences across health care systems in outcome and cost-utility of surgical and conservative treatment of chronic low back pain: a study protocol. BMC Musculoskelet Disord.

[9] Stubbs B, Koyanagi A, Thompson T (2016). The epidemiology of back pain and its relationship with depression, psychosis, anxiety, sleep disturbances, and stress sensitivity: data from 43 low- and middle-income countries. Gen Hosp Psychiatry.

[10] Casazza BA (2012). Diagnosis and treatment of acute low back pain. Am Fam Physician.

[11] Tataryn N, Simas V, Catterall T, Furness J, Keogh JW (2021). Posterior-chain resistance training compared to general exercise and walking programmes for the treatment of chronic low back pain in the general population: a systematic review and meta-analysis. Sports Med Open.

[12] Bernstein IA, Malik Q, Carville S, Ward S (2017). Low back pain and sciatica: summary of NICE guidance. BMJ.

[13] Qaseem A, Wilt TJ, McLean RM (2017). Noninvasive treatments for acute, subacute, and chronic low back pain: a clinical practice guideline from the American College of Physicians. Ann Intern Med.

[14] Shi J, Hu ZY, Wen YR (2022). Optimal modes of mind-body exercise for treating chronic non-specific low back pain: Systematic review and network meta-analysis. Front Neurosci.

[15] O'Connell NE, Cook CE, Wand BM, Ward SP (2016). Clinical guidelines for low back pain: a critical review of consensus and inconsistencies across three major guidelines. Best Pract Res Clin Rheumatol.

[16] Ganesh GS, Khan AR, Das S, Khan A (2023). Prescription of therapeutic exercise for chronic low back pain management: a narrative review. Bulletin of Faculty of Physical Therapy.

[17] Stochkendahl MJ, Kjaer P, Hartvigsen J (2018). National clinical guidelines for non-surgical treatment of patients with recent onset low back pain or lumbar radiculopathy. Eur Spine J.

[18] Ferreira PH, Ferreira ML, Maher CG, Refshauge K, Herbert RD, Hodges PW (2010). Changes in recruitment of transversus abdominis correlate with disability in people with chronic low back pain. Br J Sports Med.

[19] Dreisinger TE (2014). Exercise in the management of chronic back pain. Ochsner J.

[20] Vadalà G, Russo F, De Salvatore S, Cortina G, Albo E, Papalia R, Denaro V (2020). Physical activity for the treatment of chronic low back pain in elderly patients: a systematic review. J Clin Med.

[21] Bortz WM 2nd (1989). Redefining human aging. J Am Geriatr Soc.

[22] Şahin N, Karahan AY, Albayrak İ (2018). Effectiveness of physical therapy and exercise on pain and functional status in patients with chronic low back pain: a randomized-controlled trial. Turk J Phys Med Rehabil.

[23] Raiszadeh K, Tapicer J, Taitano L, Wu J, Shahidi B (2021). In-clinic versus web-based multidisciplinary exercise-based rehabilitation for treatment of low back pain: prospective clinical trial in an integrated practice unit model. J Med Internet Res.

[24] Li Y, Yan L, Hou L (2023). Exercise intervention for patients with chronic low back pain: a systematic review and network meta-analysis. Front Public Health.

[25] Tang S, Qian X, Zhang Y, Liu Y (2016). Treating low back pain resulted from lumbar degenerative instability using Chinese Tuina combined with core stability exercises: a randomized controlled trial. Complement Ther Med.

[26] Rydeard R, Leger A, Smith D (2006). Pilates-based therapeutic exercise: effect on subjects with nonspecific chronic low back pain and functional disability: a randomized controlled trial. J Orthop Sports Phys Ther.

[27] Cui D, Janela D, Costa F (2023). Randomized-controlled trial assessing a digital care program versus conventional physiotherapy for chronic low back pain. NPJ Digit Med.

[28] Ji G, Neugebauer V (2011). Pain-related deactivation of medial prefrontal cortical neurons involves mGluR1 and GABA(A) receptors. J Neurophysiol.

[29] Vitoula K, Venneri A, Varrassi G, Paladini A, Sykioti P, Adewusi J, Zis P (2018). Behavioral therapy approaches for the management of low back pain: an up-to-date systematic review. Pain Ther.

